# Clonal Hematopoiesis and Vascular Disease

**DOI:** 10.1007/s00281-022-00913-z

**Published:** 2022-02-04

**Authors:** Kaushik Amancherla, John A Wells, Alexander G Bick

**Affiliations:** a Division of Cardiovascular Medicine, Department of Medicine, Vanderbilt University Medical Center, Nashville, TN, USA; b Division of Genetic Medicine, Department of Medicine, Vanderbilt University Medical Center, Nashville, TN, USA

## Abstract

Somatic mutations in hematopoietic stem cells are common with aging and can result in expansion of clones harboring mutations, termed clonal hematopoiesis. This results in an increased risk of blood cancers, but has also been linked with chronic inflammatory disease states. In recent years, clonal hematopoiesis has been established to have a causative role in atherogenesis and cardiovascular disease. Additionally, as the effector cells have been identified to be immune cells, there is ongoing interest in assessing whether dysregulated immune function plays a role in other chronic inflammatory conditions such as rheumatologic disease. Here we summarize current understanding of clonal hematopoiesis with a focus on cardiovascular disease and inflammation while outlining the potential, yet unexplored, relationship between clonal hematopoiesis and autoimmune disease.

## Graphical abstract.

Hematopoietic stem cells (HSCs) continually regenerate blood cells. Acquisition of a somatic mutation that provides a selective advantage, a driver mutation, can result in clonal expansion. Clonal hematopoiesis of indeterminate potential, where somatic mutations in certain cancer-associated genes result in clonal expansion in the absence of overt malignancy, can result in atherosclerotic cardiovascular disease in multiple vascular beds, inflammation, and may also contribute to the pathogenesis of autoimmune disease. Many questions remain unanswered regarding the relationship between clonal hematopoiesis and inflammatory disorders.

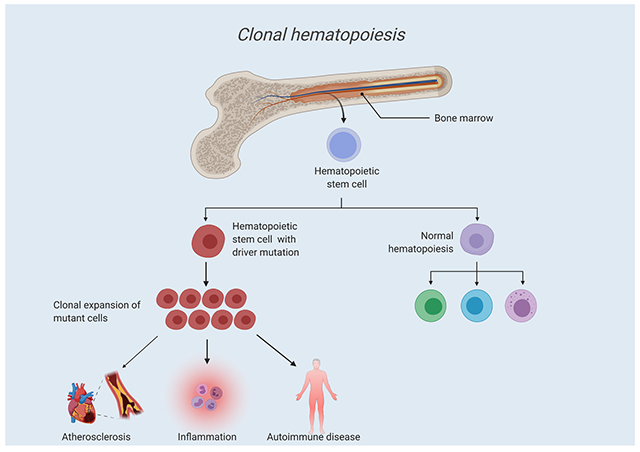

## Introduction

Clonal hematopoiesis has emerged as a powerful link between aging and inflammation. The prevalence of clonal hematopoiesis increases significantly with age^1^ and occurs in a variety of organs^2^. Through self-renewing hematopoietic stems cells (HSCs), the bone marrow continuously regenerates cells of the blood in order to maintain homeostasis^3^. Over time, DNA damage or errors in DNA replication result in the acquisition of somatic mutations in HSCs. It is estimated that HSCs acquire approximately 14 mutations per year^4^. The majority of these mutations are of no consequence but certain mutations, typically ones found in hematological malignancies, can result in a selective advantage and lead to expansion of a clone^5^. By the age of 70, more than 10% of individuals harbor clones that make up an appreciable fraction of peripheral blood^1^. Initial studies of clonal hematopoiesis associated with aging demonstrated a skewed pattern of X-chromosome inactivation in a large proportion of healthy women over the age of 60 years^6^. Since then, advancements in sequencing platforms and computational techniques have led to improved understanding of age-related clonal skewing.

Clonal hematopoiesis of indeterminate potential (CHIP) is defined as the presence of mutations in leukemia-driver genes in the absence of hematologic malignancy when the mutations are present at a variant allele fraction (VAF) of ≥2%. The term “indeterminate potential” reflects the finding that most individuals harboring these mutations do not go on to develop blood cancers. The VAF cut-off of 2% is relatively arbitrary and previously criticized for being based on limits of sequencing technologies, but has borne out to have practical implications as the presence of clones at low VAF does not seem to have an association with subsequent progression to leukemia^7,8^. The most commonly mutated genes associated with CHIP include *DNMT3A, TET2,* and *ASXL1*, with variability noted in the distribution of CHIP mutations across the spectrum of age^9,10^ and in the risk associated with progression to hematologic malignancy^11,12^. As these mutations occur in genes associated with the development of blood cancers, those who harbor CHIP mutations are at higher risk of hematologic malignancy^1,13^. There is an approximate 0.5-1% annual risk of progression to hematologic neoplasm, such as myelodysplastic syndrome or acute myeloid leukemia^1,13,14^.

Surprisingly, persons carrying CHIP mutations were also noted to be at a much higher risk for increased overall mortality than could be explained solely by progression to hematological malignancy^1^. Subsequent studies have raised the possibility that increased risk of cardiovascular disease, such as coronary artery disease, peripheral arterial disease, stroke, and heart failure, could help explain this increased mortality^1,15–17^. These investigations have further elucidated the mechanisms by which CHIP mutations may play causal roles in the development of atherosclerotic cardiovascular disease (ASCVD). In subsequent sections, we will discuss the biology of CHIP mutations as they relate to ASCVD and inflammation (**Graphical Abstract**).

## The biology of CHIP

Genes that have been implicated in CHIP typically encode proteins involved in DNA modifications and the majority of mutations have been consistently found in *DNMT3A*, *TET2*, and *ASXL1*. *DNMT3A* is a DNA methyltransferase that plays a critical role in HSC differentiation and is thought to function as a tumor suppressor gene^18^. Similarly, *TET2* is a tumor suppressor gene, while less is understood about *ASXL1*. The products of other genes, such as *PPMD1* and *TP53* respond to cellular stress and play key roles in the DNA damage response, while JAK2 is involved in cell differentiation, proliferation, and cytokine signaling pathways^19^. The Table highlights major genes associated with CHIP mutations and the functions of their gene products.

## Clonal hematopoiesis and inflammation

As HSCs give rise to circulating immune cells, mutations associated with CH are also present in these cells and impact immune function. However, the distribution of CHIP mutations varies between different immune cell types. Mutational VAFs are higher in circulating monocytes, granulocytes, and natural killer cells in comparison to B- and T-cells^20^. Different somatic mutations are found in different frequencies amongst these cell types, with mutations more commonly found in the myeloid cells and natural killer cells. Studies have shown that T cells harboring *DNMT3A* and *TET2* loss-of-function mutations have dysregulated T-cell function^21,22^. T cells lacking *Dnmt3a* and *Tet2* show alterations in polarization and promotes inflammation by affecting both T helper cells and regulatory T cells^23–25^. Less is known about the role of CHIP in B cell function.

Several CHIP-associated mutations play key roles in modulating immune responses. Ten-eleven-translocation-2, or *TET2*, is a methylcytosine dioxygenase involved in regulation of transcription and is the second most frequently mutated gene in CHIP^10^. Recent studies have identified that the loss of *TET2* in mice predisposed to atherosclerosis due to knockout of the low-density lipoprotein receptor (*Ldlr*) express higher levels of proinflammatory genes, such as *Il1b, Il6, Cxcl1, Cxcl2*, and *Cxcl3*^16,26^. In particular, the loss of Tet2 results in upregulation of interleukin-6 (IL-6) production which is rescued by wild-type Tet2^27^. Furthermore, as will be discussed in subsequent sections, increased activation of the NLRP3 inflammasome in Tet2-deficient macrophages results in increased production of IL-1β^26,28^. Similar, but not identical, gene expression patterns have also been identified in macrophages with *Dnmt3a* mutations^29^.

## Clonal hematopoiesis and cardiovascular disease

As previously discussed, early studies linked CHIP with both coronary artery disease and early-onset myocardial infarction^1,16^. The presence of CHIP mutations was associated with an approximate two-fold increased risk for development of coronary artery disease. Jaiswal et al.^16^ initially recognized that the presence of CHIP mutations was associated with increased risk of incident coronary artery disease and stroke. A subsequent study replicated these findings in additional case-control cohorts^16^. In that study, CHIP carriers with VAF >10% were also more likely to develop coronary artery disease in comparison to those harboring smaller clone size.

Experimental evidence has now confirmed that CHIP mutations have a causal relationship with cardiovascular disease. An investigation involving single-cell RNA sequencing identified increased expression of pro-inflammatory genes in peripheral monocytes and T cells in patients harboring *DNMT3A* mutations^30^. While prior studies have shown increased expression of IL-1β and IL-6 with *Dnmt3a* mutations, this mutation has been less extensively studied in its relationship to ASCVD^29^. In contrast, two groups recently interrogated how mutations in *TET2* contribute to the development of atherosclerosis^16,26^. Using *Tet2^−^/^−^* mice and transferring their bone marrow to that of *Ldlr^−^/^−^* mice, which are prone to atherosclerosis due to knockout of the LDL receptor, Jaiswal et al.^16^ showed that mice that had the *Tet2*-deficient bone marrow had significantly larger atherosclerotic plaques in the aorta despite having a similar fasting serum lipid profile as control mice. Fuster et al.^26^ independently utilized the same mouse model and further characterized that *TET2*-deficient macrophages exhibited increased secretion of interleukin-1β (IL-1β) through increased expression of the NLRP3 inflammasome. The difference in aortic plaque size between the *Tet2*-deficient mice and control mice was abrogated by use of an NLRP3 inhibitor, identifying a key role for NLRP3-mediated IL-1β overproduction in promoting atherosclerosis in those harboring *TET2* CHIP mutations. In addition to IL-1β, both studies also showed that *TET2* deficiency leads to increased expression of proinflammatory cytokines and chemokines in macrophages, especially IL-6. A separate group evaluated the mechanisms through which clonal hematopoiesis due to *JAK2^V617F^* mutations contribute to atherogenesis in the background of *Ldlr^−^/^−^* mice and found altered inflammatory responses by macrophages leading to larger necrotic core formation in plaques^31^. It was subsequently found that the *JAK2^V617F^* mutation led to increased expression of the AIM2 inflammasome and that *Aim2*-deficiency ameliorated atherosclerotic burden^32^. These studies highlight the central roles of the NLRP3 and AIM2 inflammasomes, IL-1β, and IL-6 in mediating the effects of certain CHIP mutations. Less is known about the association between less common CHIP mutations and ASCVD.

While the relationship between CHIP and coronary artery disease has been most studied, recent data suggest that this association extends to the entire arterial system. Zekavat et al.^33^ identified that the presence of CHIP mediated by mutations in the DNA damage response gene, *TP53*, was associated with a 1.7-fold increase in incident peripheral arterial disease (PAD). In the *Ldlr* knockout model, p53 knockout resulted in accumulation of p53-deficient macrophages in aortic plaque in an IL-6- and IL-1β-independent manner. Unlike *TET2* mutations, *TP53* CHIP mutations resulted in proliferation of p53-deficient macrophages within atherosclerotic plaques and accelerating the development of atherosclerosis. The association between CHIP mutations and atherosclerosis across several vascular beds, including the cerebral, renal, and mesenteric vasculature, was also observed. Notably, in addition to identifying a different mechanism through which CHIP mutations contribute to atherogenesis, this study also offered a link between cytotoxic chemotherapy, which often results in mutations in DNA damage response genes (such as *TP53*), and subsequent risk of ASCVD.

## Clonal hematopoiesis and autoimmune disease

As several studies have shown an association between CHIP mutations and a proinflammatory profile, it would seem reasonable that CHIP may play a role in autoimmune disorders. However, there is a paucity of studies that have evaluated the role of CHIP in autoimmune disease. Savola et al^34^ evaluated blood samples of 59 patients with rheumatoid arthritis and did not find an increased rate of clonal hematopoiesis. Additionally, there was no relationship between CHIP and severity of rheumatoid arthritis disease activity. In contrast, CHIP was present in 30.4% of patients with anti-nuclear cytoplasmic antibody (ANCA)-associated vasculitides^35^.

However, this study lacked a control group, but rather, compared the results with reported prevalence of CHIP in other cohorts of similar age which obfuscates whether the presence of CHIP is associated with an increased risk of autoimmune vasculitis. These studies are hampered by small sample sizes and, as mentioned previously, CHIP mutations are much more common in myeloid cells whereas T cells and B cells are thought to play prominent roles in autoimmune disease. Future studies may focus on populations of lymphoid CHIP (L-CHIP) cells. These clones have been shown to increase the risk of lymphoid neoplasms. However, a connection between L-CHIP variants and autoimmune disease has not yet been established^36^.

## Clinical implications of clonal hematopoiesis

The discovery of CHIP and its connection to cardiovascular end-points suggests that modulating inflammation via targeted treatment may have significant implications in the clinical management of patients. While the role of plasma lipids in promoting atherosclerosis had been established, prior experimental and clinical data also suggested a critical role for inflammation in atherogenesis^37^. The Canakinumab Antiinflammatory Thrombosis Outcome Study (CANTOS) directly tested the inflammatory hypothesis of atherothrombosis by administering canakinumab, an IL-1β antagonist that does not affect serum lipid levels, to patients with prior myocardial infarction and persistently elevated high-sensitivity C-reactive protein (hsCRP), a marker of inflammation^38^. Canakinumab led to a significant reduction in the primary endpoint of first occurrence of nonfatal myocardial infarction, any nonfatal stroke, or cardiovascular death in a dose-dependent manner along with a reduction in a key secondary endpoint of unstable angina that required urgent revascularization. Notably, and as expected, with modulation of proinflammatory responses, there was a significant increase in deaths attributed to infection or sepsis in the canakinumab arm.

In a finding particularly relevant to CHIP, as experimental evidence had identified IL-1β-mediated inflammation as a key driver of atherosclerosis in animal models of CHIP, a secondary analysis of the CANTOS trial of patients sought to assess the relationship between CHIP mutations and response to canakinumab^39^. Approximately 9% of patients in the trial (n = 344) were carriers of CHIP, with *TET2* mutations being the most common. Unsurprisingly, patients harboring CHIP, particularly *TET2* mutations, had a greater treatment response to canakinumab compared to patients without CHIP (hazard ratio = 0.36, *p-value* = 0.034) suggesting that targeting IL-1β signaling specifically in certain CHIP carriers may significantly ameliorate the development of cardiovascular disease^39^.

Mendelian randomization can be utilized to study whether genetic variants that mimic the effects of an exposure, such as treatment with a drug, influence an outcome of interest. As discussed earlier, IL-6 is an important mediator of the effects of CHIP. Bick et al.^40^ investigated whether a common mutation of the IL-6 receptor (*IL6R*) reduces the risk of incident cardiovascular disease. The mutation, *IL6R* p.Asp358Ala, results in decreased expression of the membrane-bound form of the IL-6 receptor and represents a genetic proxy for IL-6 inhibition (similar to that of the monoclonal antibody, tocilizumab). By leveraging whole-exome sequencing of individuals in the UK Biobank, CHIP carriers who also harbored the *IL6R* p.Asp358Ala mutation had significant attenuation of incident cardiovascular disease. This represents an opportunity for future clinical trials to test whether IL-6 inhibition (for example, with tocilizumab) would minimize the risk of ASCVD in CHIP carriers. Importantly, IL-1β expression has numerous pro-inflammatory effects in addition to increasing smooth muscle cell expression of IL-6^41^. It is hypothesized that targeting IL-6 may protect against ASCVD, while avoiding the infectious complications associated with IL-1β inhibition seen in the CANTOS trial.

## Future directions

Clonal hematopoiesis is now an established independent risk factor for the development of ASCVD and portends a poor prognosis in a number of other diseases. However, many questions remain unanswered. It is yet unclear what the role of CHIP in lymphoid cells is. This is particularly relevant for autoimmune diseases that are driven by lymphoid cells rather than myeloid cells, which have been most well-studied. Investigations focused on lymphocytes may yield novel insights in the different mechanisms of CHIP and what kind of influence it has on driving lymphocyte-mediated inflammatory disease.

Another avenue to explore is the contribution of chronic inflammation in autoimmune disease to the development of CHIP. Recent studies addressed the question of whether certain environmental pressures, such as chronic inflammation secondary to infection, result in clonal expansion after initial acquisition of a somatic mutation. In chimeric mice harboring both wild-type HSCs and *Dnmt3a*-deficient HSCs, chronic mycobacterial infection resulted in significant clonal expansion^42^. Additional experimentation found that interferon gamma (IFNγ) signaling plays a key role in driving *Dnmt3a* clonal expansion. Since IFNγ has a central role in initiation and propagation of autoimmune disease^43^ and autoimmune disorders have long been associated with a significantly higher risk of cardiovascular disease and atherosclerosis^44^, longitudinal analyses of clonal expansion in patients with rheumatologic disease may provide insights into mechanisms linking the two. This, in turn, can help identify patients who would benefit most from anti-inflammatory therapy for cardiovascular protection.

There remains much to be learned in understanding the relationship between inflammation, rheumatologic disease, and clonal hematopoiesis. The use of large biobanks can help detect rational associations between CHIP and various disease states, allowing for new avenues of biological investigation and identify patients at highest risk of developing cardiovascular disease, for example. It is clear that CHIP is a prevalent phenomenon that drives inflammation, which subsequently increases atherothrombotic risk. However, further mechanistic studies are warranted to better understand how CHIP mutations modulate the immune system and to identify novel therapeutic targets.

## Figures and Tables

**Table.** Common mutations associated with clonal hematopoiesis.

Gene	Function	Cardiovascular Disease Associations
***DNMT3A***	DNA methyltransferase involved in epigenetic regulation.	Accelerated atherosclerosis; early-onset myocardial infarction; worse outcomes in heart failure, aortic stenosis.
***TET2***	Catalyzes conversion of methylcytosine to 5-hydroxymethylcytosine involved in epigenetic regulation.	Accelerated atherosclerosis; early-onset myocardial infarction; pulmonary arterial hypertension.
***ASXL1***	Plays role in chromatin remodeling and epigenetic regulation.	Early-onset myocardial infarction.
***PPM1D***	Negative regulator of TP53 tumor suppressor	Non-ischemic cardiomyopathy.
***JAK2***	Cytosolic tyrosine kinase. Component of JAK/STAT signaling cascade.	Accelerated atheroscerlsosis; enhanced thrombogenesis.
***SF3B1***	Component of the spliceosome.	Not well studied.
***SRSF2***	Component of the spliceosome.	Not well studied.
***TP53***	Tumor suppressor involved in DNA damage response pathways.	Peripheral arterial disease.
